# Fine-tuning tumor- and allo-immunity: advances in the use of immune checkpoint inhibitors in kidney transplant recipients

**DOI:** 10.1093/ckj/sfae061

**Published:** 2024-03-09

**Authors:** Tess Van Meerhaeghe, Naoka Murakami, Alain Le Moine, Sophie Brouard, Ben Sprangers, Nicolas Degauque

**Affiliations:** Departement of Nephrology, Hôpital Erasme, Université Libre de Bruxelles, Brussels, Belgium; Nantes Université, INSERM, Center for Research in Transplantation and Translational Immunology (CR2TI), UMR 1064, Nantes, France; Division of Renal Medicine, Department of Medicine, Brigham and Women's Hospital, Boston, USA; Harvard Medical School, Boston, USA; Departement of Nephrology, Hôpital Erasme, Université Libre de Bruxelles, Brussels, Belgium; Nantes Université, INSERM, Center for Research in Transplantation and Translational Immunology (CR2TI), UMR 1064, Nantes, France; Biomedical Research Institute, Department of Immunology and Infection, UHasselt, Diepenbeek, Belgium; Department of Nephrology, Ziekenhuis Oost Limburg, Genk, Belgium; Nantes Université, INSERM, Center for Research in Transplantation and Translational Immunology (CR2TI), UMR 1064, Nantes, France

**Keywords:** allograft rejection, cancer, immune checkpoint inhibitors, kidney transplantation

## Abstract

Cancer is a common complication after kidney transplantation. Kidney transplant recipients (KTR) have a 2- to 4-fold higher risk of developing cancer compared to the general population and post-transplant malignancy is the third most common cause of death in KTR. Moreover, it is well known that certain cancer types are overrepresented after transplantation, especially non-melanoma skin cancer. Immune checkpoint inhibitors (ICI) have revolutionized the treatment of cancer, with remarkable survival benefit in a subgroup of patients. ICI are monoclonal antibodies that block the binding of specific co-inhibitory signaling molecules. Cytotoxic T lymphocyte-associated antigen-4 (CTLA-4), programmed cell death protein 1 (PD-1), and its ligand programmed cell death ligand 1 (PD-L1) are the main targets of ICI. Solid organ transplant recipients (SOTR) have been excluded from clinical trials owing to concerns about tumor response, allo-immunity, and risk of transplant rejection. Indeed, graft rejection has been estimated as high as 48% and represents an emerging problem. The underlying mechanisms of organ rejection in the context of treatment with ICI are poorly understood. The search for restricted antitumoral responses without graft rejection is of paramount importance. This review summarizes the current knowledge of the use of ICI in KTR, the potential mechanisms involved in kidney graft rejection during ICI treatment, potential biomarkers of rejection, and how to deal with rejection in clinical practice.

## INTRODUCTION

Kidney transplantation is the treatment of choice for patients suffering from end-stage kidney disease, leading to better survival and quality of life compared to dialysis. Long-term outcomes in kidney transplantation have improved significantly due to better pre-transplantation matching techniques, improved surgical techniques, surveillance, and management of infectious and cardiovascular complications [1]. However, this prolonged survival is at the expense of an increased prevalence of cancer in kidney transplant recipients (KTR). The cumulative incidence of cancer rises according to the years after transplantation and reaches >25% after 20 years of transplantation [2]. Moreover, some cancer types are overrepresented in KTR with the greatest standardized incidence ratio observed for Kaposi sarcoma, lip cancer, and non-melanoma skin cancer (NMSC) [2]. Importantly, NMSC has a more aggressive behavior with an increased risk of metastasis and death in KTR. Transplantation as an independent risk factor may negatively affect survival for different cancer types [3]. Factors associated with increased cancer risk are older age, male gender, white ethnicity, past medical history of smoking, a longer time on dialysis, and a previous history of cancer. It is also well recognized that the type, duration, and dose of immunosuppression, higher panel reactive antibody score, higher number of HLA-DR mismatches, and deceased organ donors are associated with an increased cancer risk [4]. Immune checkpoint inhibitors (ICI) have revolutionized the treatment of different cancer types, with remarkable survival benefit in a subgroup of patients [5, 6]. Solid organ transplant recipients (SOTR) have been excluded from clinical trials owing to concerns about tumor response, allo-immunity, organ rejection, and concomitant immunosuppressive therapy. Since the indications of ICI are expected to expand, it is important to determine the risk-benefit ratio of the use of ICI in patients with SOTR.

### Immune checkpoints inhibitors

**Figure 1:**
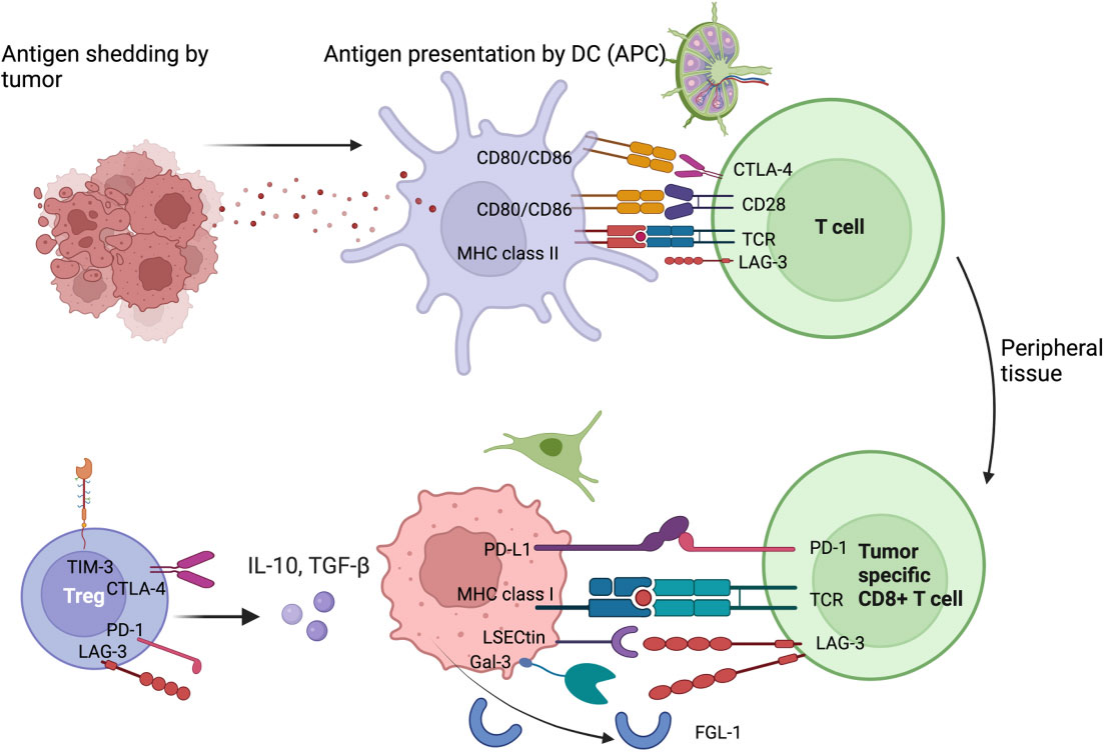
Tumor-associated antigens are presented by APC in secondary lymphoid organs to T cells. Activation of T cells is tightly regulated by immune checkpoint of which CTLA-4 in the most important proximally. In peripheral tissues and at tumoral level PD-1/ PD-L1 pathways exert an inhibitory role on T cells and promotes cancer survival. Tumoral cells also express liver sinusoidal endothelial cell lectin (LSECtin), Galectin-3 (Gal-3), and fibrogen-like protein-1 (FGL-1) that bind LAG-3 to induce T cell anergy. Tumor survival and growth is further enhanced by the presence of tumor-infiltrating regulatory T cells that are known to express higher levels of CTLA-4, PD-1, LAG-3, and T cell immunoglobulin and mucin domain-containing protein 3 (TIM-3) and to secrete higher levels of IL-10 and TGF-beta, promoting tumoral tolerance within the tumoral microenvironment.

T lymphocytes are the critical players in antitumoral response and allograft rejection. The T cell activation process involves antigen presentation by major histocompatibility complex (MHC) molecules on the antigen-presenting cells (APCs) or tumor cells to the T cell receptor (TCR) on T cells (Fig. 1). Following engagement of the TCR with cognate antigen, CD28 provides the necessary second (co-stimulatory) signal for T cell activation by binding to CD80 (B7-[1]) and CD86 (B7-[2]) on APCs. The interaction with co-stimulatory molecules is tightly regulated by inhibitory checkpoints to avoid collateral damage and autoimmunity. Indeed, activated T cells express multiple co-inhibitory receptors such as lymphocyte-activation gene 3 (LAG-3), programmed cell death protein 1 (PD-1), and cytotoxic T lymphocyte-associated protein-4 (CTLA-4) among others [7]. Programmed cell death ligand 1 (PD-L1), the primary ligand of PD-1, is expressed on different cell types, including T cells, B cells, tumor cells, and tumor-infiltrating myeloid cells. Interaction of PD-1 with PD-L1 on tumor cells induces T cell exhaustion within the tumoral environment (TME), maintains immune tolerance, and favors tumor escape. Relative to PD-1, CTLA-4 acts proximally at the T cell priming sites and limits the extent of T cell activation in secondary lymphoid organs. CTLA-4 also plays a prominent role in the regulation of regulatory T cells (Treg) within the TME [8–11]. LAG-3 is a co-inhibitory molecule expressed by cytotoxic CD8^+^ T cells and Treg. LAG-3 principally interacts with MHC class II expressed on APC but can also interact with liver sinusoidal endothelial cell lectin, Fibrogen-like protein–1 and galectin-3. Binding of LAG-3 to its ligands leads to inhibition of T cell proliferation, decreased cytokine production, and cytolytic function [12].

ICI are monoclonal antibodies directed against the immune checkpoints of the immune system. To date, eight ICI have been approved for 17 different malignancies (Table 1) [11]. Moreover, a LAG-3 inhibitor, Relatlimab was recently approved for the treatment of advanced melanoma [13]. ICI are one of the core pillars in the treatment of cancer today, but its usage in SOTR is limited. Indeed, immunosuppressive medications act in direct opposition to ICI, which are used to enhance the adaptive immune response toward cancer antigens. On the other hand, activation of the immune system may lead to an enhanced immune reactivity including autoimmunity and allo-immunity with a greater risk of allograft rejection.

**Table 1:** FDA approved ICI therapy and their current indications. Adapted from Wang *et al.* [73].

Drug	Target	Date of approval by the FDA	Indications
Ipilimumab	CTLA-4	2011	Melanoma, renal cell carcinoma, colorectal cancer, hepatocellular carcinoma, non-small cell lung cancer, malignant pleural mesothelioma, esophageal cancer
Nivolumab	PD-1	2014	Melanoma, non-small cell lung cancer, malignant pleural mesothelioma, renal cell carcinoma, classical Hodgkin lymphoma, squamous cell carcinoma of the head and neck, urothelial carcinoma, colorectal cancer, hepatocellular carcinoma, esophageal cancer, gastric cancer, gastroesophageal junction cancer, esophageal adenocarcinoma
Pembrolizumab	PD-1	2014	Melanoma, non⁠-small cell lung cancer, head and neck squamous cell carcinoma, classical Hodgkin lymphoma, primary mediastinal large B cell lymphoma, urothelial carcinoma, non-muscle invasive bladder cancer, colorectal cancer, gastric cancer, esophageal cancer, cervical cancer, hepatocellular carcinoma, Merkel cell carcinoma, renal cell carcinoma, endometrial carcinoma, cutaneous squamous cell carcinoma, triple-negative breast cancer
Atezolizumab	PD-L1	2016	Non-small cell lung cancer, small cell lung cancer, hepatocellular carcinoma, melanoma, alveolar soft part sarcoma
Durvalumab	PD-L1	2017	Non-small cell lung cancer, small cell lung cancer, biliary tract cancer, hepatocellular carcinoma
Avelumab	PD-L1	2017	Merkel cell carcinoma, urothelial carcinoma, renal cell carcinoma
Cemiplimab	PD-1	2019	Cutaneous squamous cell carcinoma, basal cell carcinoma, non-small cell lung cancer
Dostarlimab	PD-1	2021	Endometrial cancer
Relatlimab	LAG-3	2022	Melanoma

### Current data on the use of immune checkpoint inhibitors in kidney transplant recipients

The use of ICI in SOTR and KTR is mainly based on case reports, case series, and systematic reviews of the literature [14–21]. Most patients in the published reports were treated with anti-PD-1, were suffering from metastatic melanoma and were started on ICI with a mean of 9 years after transplantation. The rate of rejection is highest among KTR compared to liver, heart, and lung transplant patients and ranges from 41 to 48%. A common feature in the different case reports and case series published is the aggressiveness of the acute allograft rejection under ICI. Most papers report high levels of allograft loss (up to 83%) after rejection, with higher mortality for heart and liver transplants compared to KTR. This is mainly based on the fact that KTR can be hemodialyzed when graft loss occurs [14, 15]. When biopsies have been performed, histological analysis reveals mostly pure T cell-mediated rejection (TCMR) or mixed T cell and antibody-mediated rejection (ABMR). Although different regimens have been proposed ranging from corticosteroids, intravenous immunoglobulins, to thymoglobulin, and ultimately transplantectomy [15, 18], no effective treatment has been reported and response rates are poor.

Factors associated with graft rejection are a previous history of rejection, treatment with low-dose prednisone (<10 mg per day), the use of anti-PD-1 compared to anti-CTLA-4 or anti-PD-L1, and combination therapy (Table 2). Within the different types of anti-PD-1 used, pembrolizumab has the highest rejection rate, compared to nivolumab and cemiplimab [18]. Time after transplantation of at least 8 years, treatment with at least two immunosuppressive drugs, and/or an mTOR inhibitor-based regimen and grafts from deceased kidney donors are associated with a lower rejection rate after ICI treatment in KTR. Interestingly, patients suffering from cutaneous squamous cell carcinoma (cSCC) have the highest cancer response rates compared to other cancer types. Rünger *et al.*, showed that SOTR suffering from cSCC have 59.4% response rates, defined as partial and complete response. Moreover, the ideal response (tumor response without graft rejection) was also highest (50%) in this subgroup of patients [20]. One small retrospective study with seven KTR suffering from advanced cSCC treated with cemiplimab demonstrated a good overall tumoral response (43%) with only one patient experiencing an allograft rejection [22]. Hanna *et al*. recently published the results of the CONTRAC-1 study. This open-label prospective study included 12 KTR suffering from advanced cSCC treated with cemiplimab. Overall response rate (ORR) was 46% (90% CI, 22 to 73) and no allograft rejection occurred during a median follow-up of 6.8 (range 0.7–29.8) months [23]. This is the first prospective study of KTR to show encouraging results concerning the use of anti-PD-1 for advanced cSCC.

**Table 2:** Characteristics of KTR with allograft rejection in the context of ICI [14, 15, 17, 18].

Risk of rejection	Up to 48%
Diagnosis	Rise in serum creatinine and kidney biopsy
Median time to graft rejection	3 weeks
Histology of rejected allograft	TCMR or mixed TCMR and ABMR
Response to treatment	Poor with up to 70% graft loss
Risk factors	Low-dose corticosteroids, history of graft rejection, anti-PD-1 treatment or combination therapy
Factors associated with lower risk of rejection	mTOR and at least two immunosuppressants at time of ICI initiation

KTR: kidney transplant recipients, TCMR: T cell-mediated rejection, ABMR: antibody-mediated rejection. PD-1: programmed cell death-1, mTOR: mammalian target of rapamycin

Most retrospective studies showed similar response rates for all cancer types in KTR compared to the general population. This should be interpreted with caution due to possible publication bias. However, the prospective, phase 1 study of Carroll *et al.* confirmed the good ORR in this patient population, but lacked a control group [24]. In a recent systematic review, it was also demonstrated that patients with an intact graft had a 1.7-fold higher tumor response rate compared to patients with graft rejection. This is a very interesting finding, but can be related to deleterious treatment with immunosuppression for allograft rejection, the premature stopping of the ICI and possibly death [20].

### Mechanisms of allograft rejection in the context of ICI

**Figure 2:**
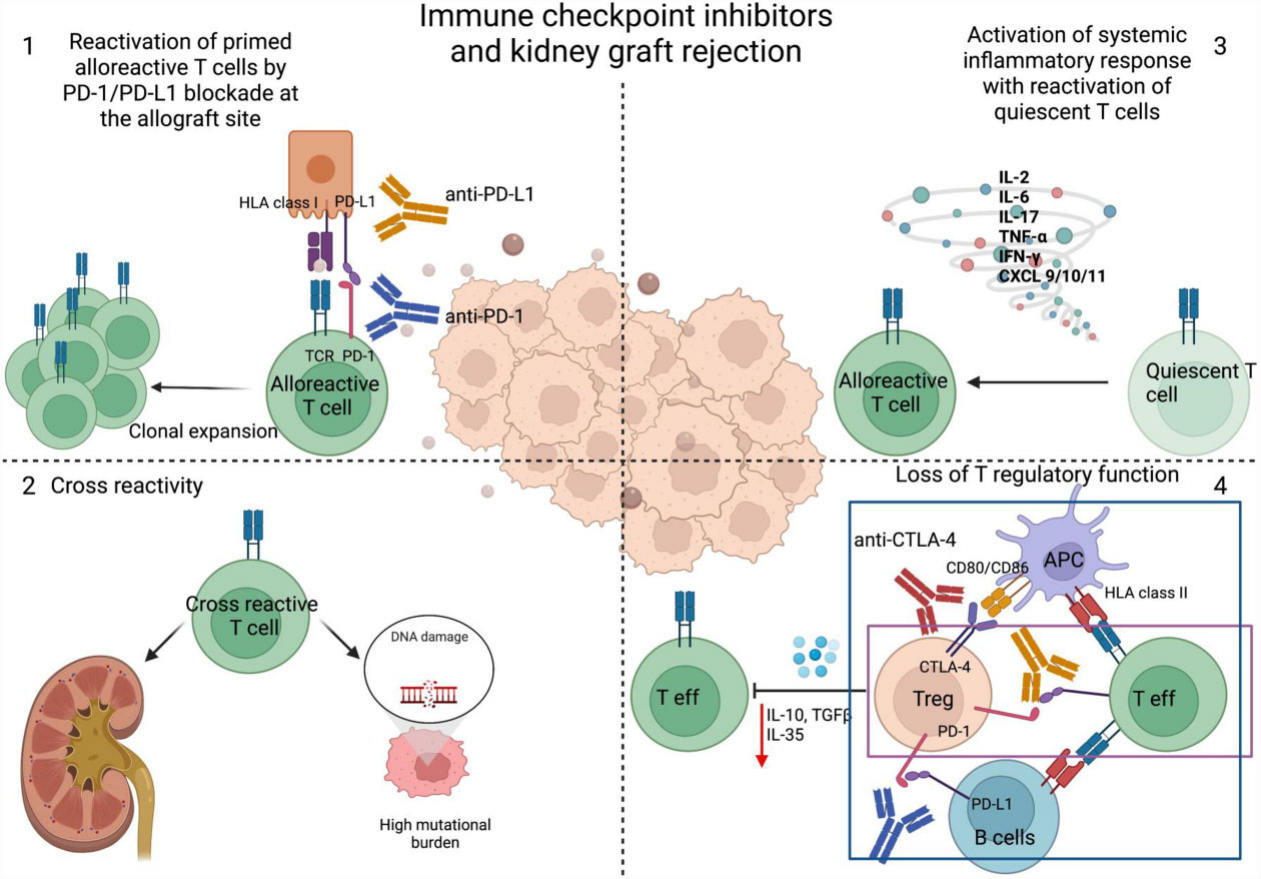
Potential mechanisms involved in kidney allograft rejection in the context of ICI. (1) Reactivation of alloreactive quiescent T cells by blocking the PD-1/PD-L1 pathway. (2) Cross-reactivity between tumoral neoantigens and kidney allograft antigens. (3) Systemic inflammation can cause overactivation of the immune system, with off-target effects and potential activation of dormant alloreactive T cells. (4) CTLA-4 expressed on Tregs interacts with co-stimulatory molecules CD80/86 preventing APCs from effectively stimulating effector T cells. CTLA-4 can also directly interact with CD80/86 expressed on effector T cells. PD-1 on Treg prevents alloreactive B cells from stimulating other T cells and can inhibit directly T effector cells expressing PD-L1. Blocking both pathways leads to loss of regulatory T cells function and activation of host alloimmune responses.

The mechanisms of allograft rejection in the context of ICI are poorly understood and not only explained by the reduction of maintenance immunosuppression as some studies show allograft rejection without prior reduction in immunosuppression (Fig. 2). First, one can imagine that quiescent alloreactive T cells are reactivated by ICI. Indeed, the PD-1/PD-L1 pathway is of utmost importance in maintaining peripheral tolerance in different experimental models of transplantation. In pre-clinical transplant models, blockade of PD-1/PD-L1 pathway *in vivo* with anti-PD-1 antibodies has been shown to lead to an accelerated rejection characterized by the expansion of alloreactive effector CD8^+^, Th1 differentiation of CD4^+^ T cells and decrease in FoxP3 CD4^+^CD25^+^ T cells infiltration in affected grafts [10, 25–27]. Interestingly, APC transfected with adenovirus coding for PD-L1 are able to enhance survival in fully mismatched kidney transplant models in rats [28] Furthermore, it is known that human tubular epithelial cells constitutively express PD-L1 and PD-L2, and that the expression can be upregulated by IFN-β and IFN-γ [29]. On a molecular level, KTR with allograft rejection have an upregulation of tissue PD-L1 mRNA compared to KTR with interstitial fibrosis/tubular atrophy or BK nephropathy [30]. This points to a potential protective effect of PD-L1 upregulation during immune activation. In mouse kidney transplant models, the PD-1/PD-L1 pathway was also important to prevent acute rejection immediately after transplantation, and thus was not only related to chronic antigen stimulation of T cells [31]. *Ex vivo* perfusion of donor kidneys with membrane-anchored-protein PD-L1 (map-PD-L1) in a rat model protected against acute kidney rejection with a reduction in T cell graft infiltration and increase in Treg [32]. However, the PD-1/PD-L1 pathway is only effective when the TCR is engaged. In a pancreatic islet allograft model treated with anti-CD3 antibody, there was a long-standing anergy of CD8^+^ T cells marked by absence of an inflammatory gene expression. The intragraft CD8^+^ T cells produced transforming growth factor β (TGFβ) and expressed PD-1 and PD-L1. TGFβ was important for the expression of the inhibitory receptors as blockade of the cytokine led to graft rejection in this model [33]. Not only the expression of PD-L1 in target tissue can protect against allograft rejection, but overexpression of PD-1 on T cells in combination with CTLA-4 blockade can promote allograft tolerance as shown in a fully MHC mismatched cardiac transplant model [34]. Recent evidence for the reactivation of pre-existing alloreactive T cells in ICI-mediated allograft rejection in a patient with melanoma was provided by Dunlap *et al.* The authors showed an expansion of circulating alloreactive CD8^+^ T cell clones that accumulated in the kidney allograft during rejection while receiving anti-PD-1 treatment, but were not present in tumor tissues. The expanded CD8^+^ T cells had a specific transcriptomic profile compatible with an elevated activation and tissue resident-memory signatures by the expression of ZNF683, CXCR3, and HLA-DR [35].

While rejection is highest under anti-PD-1 therapy, rejection is also seen with the use of anti-CTLA-4. This is not surprising as CTLA-4 is an important regulator of T cell priming in the secondary lymphoid organs and is constitutionally expressed by Treg cells. CTLA-4 knock-out mice develop severe lymphoproliferative disease and die at a young age [36]. On the contrary Belatacept, a CTLA-4Ig, is used for maintenance immunosuppression in kidney transplantation [37]. It is notable from the immune-oncology standpoint that the use of CTLA-4Ig was associated with post-transplant lymphoproliferative disease in Epstein–Barr virus-negative patients, and it is now contraindicated for CTLA-4Ig to be used in Epstein–Barr virus-negative KTR [38].

In ICI-induced allograft rejection, a cross-talk between tumor-related immune response and alloreactivity cannot be ruled out. For instance, one can imagine that KTR who reduce their tumor size but undergo allograft rejection after ICI might also develop T cell repertoires that share common antigenic specificities for tumor and allogeneic peptides. Examining TCR specificity of CD8^+^ T cells infiltrating the allograft and tumoral tissue may provide evidence of such cross-reactivity.

As parallelism with immune-related adverse events, one can imagine that alteration of the local or systemic cytokine profile can tip the balance toward inflammation leading to tissue and potential allograft damage [39]. Furthermore, the loss of T regulatory function by inhibition of CTLA-4 or PD-1/PD-L1 pathway can lead to loss of tolerance and activation of alloreactive T cells in the context of transplantation.

The tumor itself can have an immunosuppressive function on the host by releasing adenosine, prostaglandin E2 and TGFβ1 [2, 40]. Tumor shrinkage by ICI can therefore indirectly augment host responses toward the allograft.

### Biomarkers of allograft rejection in the context of ICI

Several surrogate markers predictive of either rejection or tolerance have been identified in KTR, but their ability to identify ‘high-risk’ or ‘low-risk’ patients before and during administration of ICI remains to be established [41–47]. Increasing evidence points toward the role and involvement of granzyme B expressing regulatory B cells (GZMb-Breg) and TEMRA CD8^+^ in kidney allograft survival. Based on phenotypical analyses, it has been shown that a higher proportion of Effector Memory expressing CD45RA (TEMRA) CD8^+^ T cells predict graft failure [35, 36]. On the other hand, a unique expansion of GZMb-Breg has been identified in KTR with operational tolerance and a robust B cell signature of low-risk graft failure has been identified [41, 44].

The ultimate search for noninvasive biomarkers to diagnose allograft rejection has further evolved and those that have been demonstrated to be utile are urinary mRNA levels of CXCL10, CD3$\varepsilon $, 18S rRNA, chemokine concentration of CXCL9 and CXCL10 and plasma donor-derived cell-free DNA (dd-cfDNA) measurement [47–50]. dd-cfDNA increases before the rise in plasma creatinine and is monitored for early detection of allograft rejection and/or injury in clinical practice. Levels <1% of total dd-cfDNA are associated with the absence of active rejection, but levels >1% are indicative of active rejection [51].

The use of these biomarkers in the context of allograft rejection under ICI has been poorly investigated. Moreover, allograft rejection under ICI occurs early so biomarkers of anti-allograft response or allograft injury must be detected early. Hurkmans *et al.* demonstrated in a case report, the elevation of dd-cfDNA before clinical apparent rejection under nivolumab (anti-PD-1) treatment for metastatic melanoma [52]. The same findings were observed in the preliminary results of an ongoing prospective phase I study (NCT03816332) where dd-cfDNA increases before plasma creatinine rise in two patients with allograft rejection under cemiplimab (anti-PD-1) treatment [53]. Carroll *et al.* prespecified an exploratory endpoint in their study and measured urinary CXCL-10 concentrations before each nivolumab injection. Higher levels were seen in the two patients who rejected their allograft. They concluded that baseline monitoring of CXCL-10 concentration might be an early predictor of allograft rejection [24]. These reports are of importance and indicate that at least two biomarkers (dd-cfDNA and urinary CXCL-10) can be used for early detection of rejection in KTR under ICI therapy. However, the ultimate goal is to create a risk score to predict allograft rejection before ICI therapy to better inform our patients about the risk of allograft rejection.

So far, the gold standard for diagnosing acute rejection remains kidney biopsy. Adam *et al.* analyzed gene expression profiles on kidney biopsies of patients with ICI-induced rejection versus ICI-induced interstitial nephritis and showed a significant molecular overlap. However, interferon alpha inducible protein 27 (IFI27), was identified as a potential biomarker of ICI-induced T cell mediated rejection [54]. Some authors suggest anti-PD-L1 staining of the allograft before treatment with ICI, as a positive staining could be associated with a higher risk of rejection after ICI [55–57]. However, this implicates that a biopsy is performed before treatment, with all the risks involved in this already frail population of patients.

### Strategies to prevent allograft rejection in the context of ICI

To the best of our knowledge, no guidelines nor sufficient evidence exists to support the use of specific immunosuppressants during ICI therapy [58]. It is tempting to reduce immunosuppression before introduction of ICI to increase tumoral response at the expense of a higher risk of allograft rejection. Several retrospective studies demonstrate the association of mTORi treatment and allograft preservation. A large systematic review on the subject shows that the ideal response (tumoral response without allograft rejection) is highest among patients treated with mTORi [20]. This is not surprising since mTORi have anti-proliferative effects and could uncouple tumoral and allograft responses by maintaining Treg function without impairing the number of IFN-γ secreting T cells [59].

One case report suggests the use of pre-emptive high dose corticosteroids with sirolimus during immunotherapy to prevent graft rejection, the so-called ‘dynamic immunosuppression’ [60]. The effect of this regimen was tested in the CONTRAC-1 study on 12 KTR suffering from advanced cSCC treated with cemiplimab. No patient developed an allograft rejection and the ORR remained good (45%). This regimen, however, should be tested on a larger cohort of patients before becoming the standard of care for KTR suffering from cSCC eligible for cemiplimab, nor do we know if it can be extrapolated for other cancer types [61]. By contrast, in a prospective cohort study by Schenk *et al.* on eight KTR with advanced skin cancers (melanoma, cSCC, BCC, and Merkel cell carcinoma) treated with nivolumab with or without ipilimumab the patients were maintained on the standardized immunosuppression regimen of tacrolimus (with a target trough level of 2–5 ng/ml) and prednisone 5 mg daily. Out of eight patients enrolled, two experienced allograft rejections after addition of ipilimumab to nivolumab, and the ORR remained 33% [62].

Reduction of immunosuppression before or during ICI therapy may potentially lead to a higher risk of rejection. This question was partially addressed by Carroll *et al.*, who demonstrated that nivolumab was safe and did not impair tumoral response in KTR without pre-emptive reduction in immunosuppression [24]. (Table 3) However, one must note that baseline immunosuppression in their study was already low and variable. Belatacept (CTLA-4Ig) is commonly used as immunosuppression for KTR, but it is unclear whether it could be effectively used to prevent allograft rejection in the setting of ICI use. In non-transplant setting, abatacept, another CTLA-4Ig, has been used to treat life-threatening myocarditis in patients treated with ICI [63]. and its efficacy is being actively tested in a phase 3 clinical trial (NCT05335928) [64]. IL-6 is an important inflammatory cytokine and increased levels have been associated with immune-related adverse events and poor tumoral prognosis in the context of ICI. Anti-IL-6 has been used to treat immune-related adverse events without impairing antitumoral responses [65]. Indeed, IL-6 has an important role in regulating the balance between Th17 and Treg cells [66]. To what extent these results can be extrapolated to KTR requires further investigations [67, 68]. To our knowledge, no specific recommendations for the use of these agents in KTR exist during ICI therapy. Anti-IL-6 therapy is currently being tested in randomized trials for the treatment of TCMR and ABMR [68]. It could be an interesting target to investigate in this specific patient population.

**Table 3:** Prospective clinical trials for KTR treated with ICI.

Study	Nivolumab in transplant patients	Tacrolimus and ICI	CONTRAC-1
Cancer type	Any cancers (incurable, metastatic solid tumors)	Skin cancers (melanoma, cSCC, BCC, Merkel cell carcinoma)	cSCC
Transplant	Kidney	Kidney	Kidney
ICI	Nivolumab*	Nivolumab ± Ipilimumab	Cemiplimab
Immunosuppression	Keep the same dose	Tac (2–5 ng/ml), pred 5 mg/day	mTORi + dynamic pred
Patient #	17	8	12
Rejection	2 (11.7%)	2 (25%)	0 (0%)
ORR (CR + PR)	53%	33%	45%
Registry	ANZCTR CA209-993ISR	NCT03816332	NCT03565783
Primary institution	Royal Adelaide Hospital, multicenter	Johns Hopkins Hospital, multicenter	Dana Farber Cancer Institute
	Australia	USA	USA
Reference	*Lancet Oncol* (2022)	*J Clin Oncol* (2024)	*J Clin Oncol* (2024)

cSCC: cutaneous squamous carcinoma, BCC: basal cell carcinoma, ORR: objective response rate, CR: complete response, PR: partial response, mTORi: mammalian target of rapamycin

Recent evidence from a primary liver cancer mouse model bearing a cardiac allograft showed that dual treatment with a BET protein inhibitor (JQ1) and anti-PD-L1 was associated with better tumoral response without allograft rejection. BET protein inhibition downregulates PD-L1 expression on cardiac myocytes and protected against allograft rejection [69]. These findings are encouraging, but more translational research is necessary to investigate potential use of a BET protein inhibitor in cancer and allograft response to ICI.

### Re-transplantation after complete tumoral response

A common feature of all ICI are the long-lasting, and durable responses, even in patients with metastatic solid tumors [11]. The question of re-transplantation in the context of complete tumoral response after immunotherapy is challenging. Only one case report of kidney re-transplantation has been published in the literature so far. The patient was suffering from a metastatic cSCC and was treated with pembrolizumab (anti-PD-1) with a complete tumoral response. However, the patient developed severe TCMR of his first kidney allograft with consequent graft loss. The patient remained in remission 4.5 years after anti-PD-1 treatment and was again transplanted with a kidney from a living unrelated donor. Ten months after his new transplantation there were no signs of tumoral flare nor of allograft rejection [70]. By contrast, less convincing evidence is derived from liver transplant patients treated with ICI for hepatocellular carcinoma (HCC) before liver transplantation. A recent review revealed a rejection rate of 24% (11 out of 45 patients published) with a 36% graft loss (4 out of 11 patients). After graft loss there is a high mortality in this patient population unless urgent re-transplantation is done [71]. The authors postulated that at least 6 weeks between last dose of ICI and re-transplantation should be considered. Whether these results are applicable in KTR needs to be confirmed in larger studies.

It is also unknown whether to continue or interrupt ICI treatment in patients with prolonged responses. Current data in the non-transplant population is limited and long-term treatment with ICI may be associated with the occurrence of new chronic immune-related adverse events [11]. No data are available in SOTR and we do not know how long ICI have to be maintained for metastatic disease. Acute allograft rejection is an early complication, but long-term impact of ICI in SOTR remains elusive. Prospective studies are necessary to answer these important questions as metastatic cancer has changed into a chronic disease.

## CONCLUSION

KTR are prone to develop malignancy post-transplantation. The use of ICI in KTR is associated with an increased risk of rejection, but tumor responses seem to be encouraging despite the concomitant use of immunosuppressants. We acknowledge that most data are derived from retrospective studies and case reports. One should carefully weigh the risk and benefit from immunotherapy before starting treatment. Moderate reduction in immunosuppression may be warranted before start of ICI, but at least two immunosuppressants should be used and conversion of tacrolimus to sirolimus in combination with higher dose corticosteroids may be a reasonable treatment option to prevent rejection, without impeding tumoral response. Frequent and close monitoring of kidney function is needed to detect early allograft rejection at the start of ICI treatment. Noninvasive monitoring of kidney rejection could allow us to detect patients at risk of allograft rejection before and during ICI treatment. Ideally, a risk score should be created to help us guide ICI therapies among KTR. Finally, the use of ICI in KTR, should be carefully made by a multidisciplinary team to weigh the potential benefits against the risks.

## Data Availability

No new data were generated or analyzed in support of this re- search.
